# 2,2′-Bis(methyl­ene)-3,3′-(2-thioxo-2,3-dihydro-1*H*-benzimidazole-1,3-di­yl)dipropane­nitrile

**DOI:** 10.1107/S1600536809043438

**Published:** 2009-10-31

**Authors:** Ould M’hamed Mohamed, Hedi M’rabet, Hanene Hemissi, Mohamed El Efrit

**Affiliations:** a Laboratoire de Synthése Organique et Hétérocyclique, Faculté des Sciences de Tunis, 2092 Tunisia; b Laboratoire de Chimie des Matériaux, Faculté des Sciences de Bizerte, 7021 Zarzouna Bizerte Tunisia; c Laboratoire de Synthése Organique et Hétérocyclique, Faculté des Sciences de Tunis, 2092 Tunisia

## Abstract

In the title compound, C_15_H_12_N_4_S, the benzimidazole ring is essentially planar, with a mean deviation of 0.0082 (1) Å from the least-squares plane defined by the nine constituent atoms. In the crystal, inversion dimers linked by pairs of C—H⋯N hydrogen bonds occur.

## Related literature

Benzimidazole is a potential precursor in heterocyclic chemistry and the benzimidazol-2-thione ring is present in many pharmacologically active substances, see: Hwa *et al.* (2008 ▶). For ammonium salts from Mannich adducts as precursors for the synthesis of acrylic derivatives carrying functionalized thio­methyl groups, see: M’rabet *et al.* (2009 ▶). For a related structure, see: Khan *et al.* (2008 ▶). For bond-length data, see: Allen *et al.* (1987 ▶).
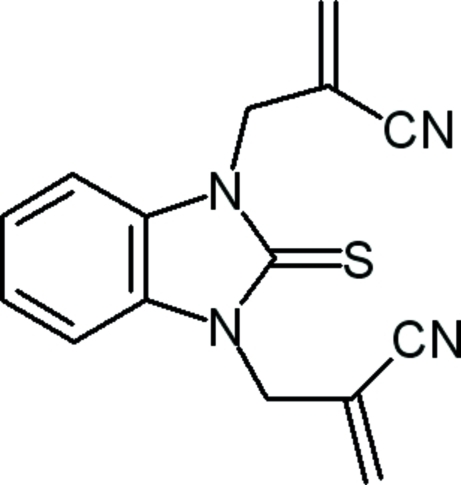


## Experimental

### 

#### Crystal data

C_15_H_12_N_4_S
*M* *_r_* = 280.35
Triclinic, 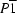

*a* = 8.6274 (3) Å
*b* = 9.8271 (2) Å
*c* = 9.8271 (2) Å
α = 70.553 (2)°
β = 89.730 (2)°
γ = 67.853 (3)°
*V* = 720.67 (3) Å^3^
*Z* = 2
Mo *K*α radiation
μ = 0.22 mm^−1^
*T* = 293 K
0.22 × 0.20 × 0.18 mm

#### Data collection

Enraf–Nonius TurboCAD-4 diffractometer
Absorption correction: none
6594 measured reflections
3297 independent reflections
2449 reflections with *I* > 2σ(*I*)
*R*_int_ = 0.018
2 standard reflections frequency: 120 min intensity decay: 3%

#### Refinement

*R*[*F* ^2^ > 2σ(*F* ^2^)] = 0.039
*wR*(*F* ^2^) = 0.111
*S* = 1.02
3297 reflections
181 parameters
H-atom parameters constrained
Δρ_max_ = 0.19 e Å^−3^
Δρ_min_ = −0.32 e Å^−3^

### 

Data collection: *CAD-4 EXPRESS* (Enraf–Nonius, 1994 ▶); cell refinement: *CAD-4 EXPRESS*; data reduction: *XCAD4* (Harms & Wocadlo, 1995 ▶); program(s) used to solve structure: *SHELXS97* (Sheldrick, 2008 ▶); program(s) used to refine structure: *SHELXL97* (Sheldrick, 2008 ▶); molecular graphics: *ORTEP-3 for Windows* (Farrugia, 1997 ▶); software used to prepare material for publication: *WinGX* (Farrugia, 1999 ▶).

## Supplementary Material

Crystal structure: contains datablocks I, global. DOI: 10.1107/S1600536809043438/pv2216sup1.cif

Structure factors: contains datablocks I. DOI: 10.1107/S1600536809043438/pv2216Isup2.hkl

Additional supplementary materials:  crystallographic information; 3D view; checkCIF report

## Figures and Tables

**Table 1:** Hydrogen-bond geometry (Å, °)

*D*—H⋯*A*	*D*—H	H⋯*A*	*D*⋯*A*	*D*—H⋯*A*
C11—H11*B*⋯N4^i^	0.93	2.51	3.387 (3)	158

Symmetry code: (i) 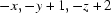.

**Fig. 1.**
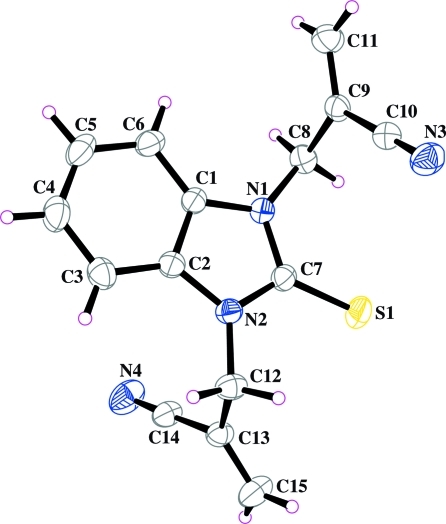
A perspective view of the molecule of (I) with displacement ellipsoids shown at the 30% probability level.

**Fig. 2.**
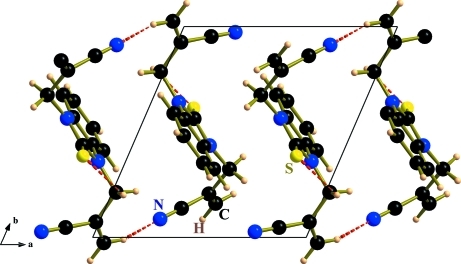
Molecular packing in the unit cell of (I) showing H-bonding interactions; H-atoms not involved in H-bonds have been excluded.

## supplementary crystallographic information

### Comment

Benzimidazole is an important scaffold in heterocyclic chemistry and benzimidazol-2-thione ring is present in many pharmacologically active substances (Hwa *et al.*, 2008). We recently showed that ammonium salts coming from Mannich adducts, constitute precursors interesting for the synthesis of acrylic derivatives carrying functionalized thiomethyle groups (M'rabet *et al.*, 2009). The results obtained encouraged us to try the action of other functionalized thiols, e.g., mercaptobenzimidazole. The analysis of the spectra for the isolated product shows that the nucleophilic attack utilized the two nitrogen atoms of the mercaptobenzimidazole whatever the stoichiometry was used. The compound was identified unequocally as *N,N*-bis(2-cyanoprop-2-enyl)benzimidazol-2-thione, (I), by the X-ray diffraction analysis.

The bond lengths and angles in the structure of (I) (Fig. 1) are in agreement with the corresponding bond lengths and angles reported for a compound closely related to (I) (Khan, H. *et al.*, 2008) and are within normal ranges (Allen *et al.*, 1987). The benzimidazole ring in (I) is essentially planar with a mean deviation of 0.0028 (1) Å from the least-squares plane defined by the nine constituent atoms. The molecular packing is stabilized by van der Waals interactions and intramolecular (C—H···S) and intermolecular (C—H···N) hydrogen bonds, which link the molecules into dimmers (Table 1 and Fig. 2).

### Experimental

To a solution of ammonium salt (12 mmol) in ethanol (50 ml), was added dropwise with stirring 10 mmol of mercaptobenzimidazole. The reaction mixture was stirred for 24 h at room temperature. The residual salt was then filtered and the solvent was removed. The obtained residue was diluted with water (20 ml) and extracted with chloroform. The organic layer was dried over MgSO_4_ and concentrated under reduced pressure. The product was chromatographed using a silica gel column with 60/40 ether/petroleum ether. The slow evaporation from the solvent afforded crystals of the title compound suitable for X-ray diffraction study.

### Refinement

All H atoms were positioned geometrically and treated as riding on the parent atoms [N–H = 0.89, C–H =0.96 Å (CH_3 _) with *U*_iso_(H) = 1.5U_eq_ and C–H = 0.96 Å (Ar–H), with *U*_iso_(H) = 1.5U_eq_].

### Crystal data

**Table:**

C_15_H_12_N_4_S	*Z* = 2
*M**_r_* = 280.35	*F*(000) = 292
Triclinic, *P*1	*D*_x_ = 1.292 Mg m^−^^3^
Hall symbol: -P 1	Mo *K*α radiation, λ = 0.71073 Å
*a* = 8.6274 (3) Å	Cell parameters from 25 reflections
*b* = 9.8271 (2) Å	θ = 9.0–11.0°
*c* = 9.8271 (2) Å	µ = 0.22 mm^−^^1^
α = 70.553 (2)°	*T* = 293 K
β = 89.730 (2)°	Prism, colourless
γ = 67.853 (3)°	0.22 × 0.20 × 0.18 mm
*V* = 720.67 (3) Å^3^	

### Data collection

**Table:**

Enraf–Nonius TurboCAD-4 diffractometer	*R*_int_ = 0.018
Radiation source: fine-focus sealed tube	θ_max_ = 28.0°, θ_min_ = 2.2°
graphite	*h* = −11→11
Nonprofiled ω scans	*k* = −12→12
6594 measured reflections	*l* = −12→12
3297 independent reflections	2 standard reflections every 120 min
2449 reflections with *I* > 2σ(*I*)	intensity decay: 3%

### Refinement

**Table:**

Refinement on *F*^2^	Primary atom site location: structure-invariant direct methods
Least-squares matrix: full	Secondary atom site location: difference Fourier map
*R*[*F*^2^ > 2σ(*F*^2^)] = 0.039	Hydrogen site location: inferred from neighbouring sites
*wR*(*F*^2^) = 0.111	H-atom parameters constrained
*S* = 1.02	*w* = 1/[σ^2^(*F*_o_^2^) + (0.0547*P*)^2^ + 0.1378*P*] where *P* = (*F*_o_^2^ + 2*F*_c_^2^)/3
3297 reflections	(Δ/σ)_max_ = 0.002
181 parameters	Δρ_max_ = 0.19 e Å^−^^3^
0 restraints	Δρ_min_ = −0.32 e Å^−^^3^

### Special details

**Table:**

Geometry. All e.s.d.'s are estimated using the full covariance matrix. The cell e.s.d.'s are taken into account individually in the estimation of e.s.d.'s in distances, angles and torsion angles; correlations between e.s.d.'s in cell parameters are only used when they are defined by crystal symmetry. An approximate (isotropic) treatment of cell e.s.d.'s is used for estimating e.s.d.'s involving l.s. planes.
Refinement. Refinement of *F*^2^ against ALL reflections. The weighted *R*-factor *wR* and goodness of fit *S* are based on *F*^2^, conventional *R*-factors *R* are based on *F*, with *F* set to zero for negative *F*^2^. The threshold expression of *F*^2^ > σ(*F*^2^) is used only for calculating *R*-factors(gt) *etc*. and is not relevant to the choice of reflections for refinement. *R*-factors based on *F*^2^ are statistically about twice as large as those based on *F*, and *R*- factors based on ALL data will be even larger.

### Fractional atomic coordinates and isotropic or equivalent isotropic displacement parameters (Å^2^)

**Table:**

	*x*	*y*	*z*	*U*_iso_*/*U*_eq_	
S1	0.21056 (7)	0.61386 (6)	0.54777 (5)	0.06747 (18)	
N1	0.12971 (16)	0.64019 (15)	0.80813 (13)	0.0438 (3)	
N2	0.36099 (16)	0.43553 (15)	0.82525 (13)	0.0443 (3)	
C2	0.33686 (19)	0.43018 (18)	0.96681 (16)	0.0429 (3)	
C1	0.19084 (19)	0.55980 (18)	0.95584 (16)	0.0421 (3)	
C7	0.2340 (2)	0.56319 (18)	0.72744 (16)	0.0452 (3)	
C9	0.01720 (18)	0.92638 (18)	0.74111 (16)	0.0447 (3)	
C10	0.1474 (2)	0.95298 (19)	0.65727 (17)	0.0478 (4)	
C14	0.3569 (2)	0.1382 (2)	0.84115 (19)	0.0529 (4)	
C13	0.45594 (19)	0.20653 (18)	0.74643 (17)	0.0455 (3)	
C12	0.5016 (2)	0.3244 (2)	0.78361 (19)	0.0510 (4)	
H12A	0.5942	0.2689	0.8632	0.061*	
H12B	0.5405	0.3823	0.7000	0.061*	
N3	0.2515 (2)	0.9733 (2)	0.59254 (18)	0.0649 (4)	
C6	0.1282 (2)	0.5886 (2)	1.07808 (18)	0.0529 (4)	
H6	0.0298	0.6752	1.0707	0.063*	
C8	−0.0187 (2)	0.78626 (19)	0.74714 (19)	0.0510 (4)	
H8A	−0.1083	0.7813	0.8060	0.061*	
H8B	−0.0575	0.7988	0.6495	0.061*	
N4	0.2784 (2)	0.0869 (2)	0.9192 (2)	0.0810 (5)	
C3	0.4283 (2)	0.3241 (2)	1.10096 (18)	0.0563 (4)	
H3	0.5264	0.2372	1.1085	0.068*	
C5	0.2197 (3)	0.4820 (2)	1.21154 (19)	0.0638 (5)	
H5	0.1814	0.4969	1.2962	0.077*	
C15	0.5064 (3)	0.1615 (3)	0.6360 (2)	0.0681 (5)	
H15A	0.4778	0.0855	0.6195	0.082*	
H15B	0.5706	0.2057	0.5745	0.082*	
C11	−0.0631 (2)	1.0256 (2)	0.8061 (2)	0.0638 (5)	
H11A	−0.0353	1.1105	0.7973	0.077*	
H11B	−0.1475	1.0105	0.8607	0.077*	
C4	0.3670 (3)	0.3534 (2)	1.22258 (19)	0.0651 (5)	
H4	0.4260	0.2853	1.3143	0.078*	

### Atomic displacement parameters (Å^2^)

**Table:**

	*U*^11^	*U*^22^	*U*^33^	*U*^12^	*U*^13^	*U*^23^
S1	0.0965 (4)	0.0682 (3)	0.0385 (2)	−0.0351 (3)	0.0141 (2)	−0.0176 (2)
N1	0.0492 (7)	0.0414 (7)	0.0399 (6)	−0.0177 (5)	0.0080 (5)	−0.0138 (5)
N2	0.0505 (7)	0.0423 (7)	0.0441 (7)	−0.0195 (6)	0.0117 (5)	−0.0193 (5)
C2	0.0511 (8)	0.0414 (8)	0.0432 (7)	−0.0239 (7)	0.0072 (6)	−0.0172 (6)
C1	0.0502 (8)	0.0432 (8)	0.0403 (7)	−0.0250 (7)	0.0090 (6)	−0.0166 (6)
C7	0.0570 (9)	0.0446 (8)	0.0419 (8)	−0.0268 (7)	0.0121 (7)	−0.0173 (6)
C9	0.0394 (7)	0.0421 (8)	0.0441 (8)	−0.0114 (6)	0.0058 (6)	−0.0107 (6)
C10	0.0486 (9)	0.0456 (8)	0.0487 (8)	−0.0170 (7)	0.0085 (7)	−0.0181 (7)
C14	0.0571 (10)	0.0575 (10)	0.0575 (9)	−0.0287 (8)	0.0212 (8)	−0.0303 (8)
C13	0.0442 (8)	0.0462 (8)	0.0505 (8)	−0.0196 (7)	0.0147 (6)	−0.0208 (7)
C12	0.0476 (8)	0.0542 (9)	0.0620 (10)	−0.0240 (7)	0.0193 (7)	−0.0295 (8)
N3	0.0637 (9)	0.0732 (11)	0.0700 (10)	−0.0339 (8)	0.0260 (8)	−0.0331 (8)
C6	0.0649 (10)	0.0564 (10)	0.0522 (9)	−0.0317 (8)	0.0190 (8)	−0.0287 (8)
C8	0.0446 (8)	0.0502 (9)	0.0538 (9)	−0.0182 (7)	0.0051 (7)	−0.0142 (7)
N4	0.0919 (13)	0.1025 (14)	0.0810 (12)	−0.0640 (12)	0.0448 (10)	−0.0434 (11)
C3	0.0618 (10)	0.0496 (9)	0.0524 (9)	−0.0208 (8)	−0.0045 (8)	−0.0136 (8)
C5	0.0945 (14)	0.0776 (13)	0.0429 (9)	−0.0515 (12)	0.0189 (9)	−0.0303 (9)
C15	0.0859 (13)	0.0850 (14)	0.0663 (11)	−0.0532 (12)	0.0376 (10)	−0.0450 (11)
C11	0.0618 (11)	0.0577 (11)	0.0732 (12)	−0.0217 (9)	0.0246 (9)	−0.0272 (9)
C4	0.0887 (14)	0.0639 (11)	0.0425 (9)	−0.0355 (11)	−0.0051 (9)	−0.0125 (8)

### Geometric parameters (Å, °)

**Table:**

S1—C7	1.6577 (15)	C9—C11	1.319 (3)
N1—C1	1.3924 (19)	C12—C13	1.503 (3)
N1—C7	1.375 (2)	C13—C14	1.434 (2)
N1—C8	1.452 (2)	C13—C15	1.317 (3)
N2—C2	1.3922 (19)	C3—H3	0.9300
N2—C7	1.367 (2)	C4—H4	0.9300
N2—C12	1.458 (2)	C5—H5	0.9300
N3—C10	1.138 (3)	C6—H6	0.9300
N4—C14	1.139 (3)	C8—H8A	0.9700
C1—C2	1.386 (2)	C8—H8B	0.9700
C1—C6	1.386 (2)	C11—H11A	0.9300
C2—C3	1.386 (2)	C11—H11B	0.9300
C3—C4	1.378 (3)	C12—H12A	0.9700
C4—C5	1.387 (4)	C12—H12B	0.9700
C5—C6	1.383 (3)	C15—H15A	0.9300
C8—C9	1.504 (3)	C15—H15B	0.9300
C9—C10	1.442 (2)		
			
C1—N1—C7	109.85 (14)	C14—C13—C15	119.89 (19)
C1—N1—C8	125.42 (14)	N4—C14—C13	177.6 (2)
C7—N1—C8	124.70 (13)	C2—C3—H3	122.00
C2—N2—C7	110.30 (14)	C4—C3—H3	122.00
C2—N2—C12	126.00 (14)	C3—C4—H4	119.00
C7—N2—C12	123.70 (13)	C5—C4—H4	119.00
N1—C1—C2	106.98 (14)	C4—C5—H5	119.00
N1—C1—C6	131.26 (16)	C6—C5—H5	119.00
C2—C1—C6	121.74 (15)	C1—C6—H6	122.00
N2—C2—C1	106.69 (13)	C5—C6—H6	122.00
N2—C2—C3	131.89 (16)	N1—C8—H8A	109.00
C1—C2—C3	121.42 (15)	N1—C8—H8B	109.00
C2—C3—C4	116.90 (18)	C9—C8—H8A	109.00
C3—C4—C5	121.62 (17)	C9—C8—H8B	109.00
C4—C5—C6	121.78 (19)	H8A—C8—H8B	108.00
C1—C6—C5	116.53 (19)	C9—C11—H11A	120.00
S1—C7—N1	127.09 (13)	C9—C11—H11B	120.00
S1—C7—N2	126.73 (13)	H11A—C11—H11B	120.00
N1—C7—N2	106.18 (12)	N2—C12—H12A	109.00
N1—C8—C9	111.91 (15)	N2—C12—H12B	109.00
C8—C9—C10	117.04 (15)	C13—C12—H12A	109.00
C8—C9—C11	124.34 (16)	C13—C12—H12B	109.00
C10—C9—C11	118.62 (16)	H12A—C12—H12B	108.00
N3—C10—C9	179.21 (18)	C13—C15—H15A	120.00
N2—C12—C13	113.07 (15)	C13—C15—H15B	120.00
C12—C13—C14	116.74 (15)	H15A—C15—H15B	120.00
C12—C13—C15	123.33 (19)		
			
C7—N1—C1—C2	0.2 (2)	C2—N2—C12—C13	101.76 (19)
C7—N1—C1—C6	−178.16 (19)	C7—N2—C12—C13	−79.4 (2)
C8—N1—C1—C2	−177.94 (16)	N1—C1—C2—N2	0.21 (19)
C8—N1—C1—C6	3.7 (3)	N1—C1—C2—C3	−179.34 (16)
C1—N1—C7—S1	179.15 (14)	C6—C1—C2—N2	178.77 (16)
C1—N1—C7—N2	−0.56 (19)	C6—C1—C2—C3	−0.8 (3)
C8—N1—C7—S1	−2.7 (3)	N1—C1—C6—C5	178.7 (2)
C8—N1—C7—N2	177.61 (15)	C2—C1—C6—C5	0.5 (3)
C1—N1—C8—C9	78.8 (2)	N2—C2—C3—C4	−179.3 (2)
C7—N1—C8—C9	−99.06 (19)	C1—C2—C3—C4	0.2 (3)
C7—N2—C2—C1	−0.6 (2)	C2—C3—C4—C5	0.7 (3)
C7—N2—C2—C3	178.92 (19)	C3—C4—C5—C6	−1.0 (4)
C12—N2—C2—C1	178.44 (16)	C4—C5—C6—C1	0.3 (4)
C12—N2—C2—C3	−2.1 (3)	N1—C8—C9—C10	59.16 (19)
C2—N2—C7—S1	−179.02 (14)	N1—C8—C9—C11	−121.4 (2)
C2—N2—C7—N1	0.69 (19)	N2—C12—C13—C14	−43.4 (2)
C12—N2—C7—S1	2.0 (3)	N2—C12—C13—C15	138.7 (2)
C12—N2—C7—N1	−178.34 (15)		

### Hydrogen-bond geometry (Å, °)

**Table:**

*D*—H···*A*	*D*—H	H···*A*	*D*···*A*	*D*—H···*A*
C8—H8B···S1	0.97	2.77	3.204 (2)	108
C11—H11B···N4^i^	0.93	2.51	3.387 (3)	158

Symmetry codes: (i) −*x*, −*y*+1, −*z*+2.
